# Systems metabolic engineering of *Corynebacterium glutamicum* for the bioproduction of biliverdin via protoporphyrin independent pathway

**DOI:** 10.1186/s13036-019-0156-5

**Published:** 2019-03-29

**Authors:** Jiho Seok, Young Jin Ko, Myeong-Eun Lee, Jeong Eun Hyeon, Sung Ok Han

**Affiliations:** 1 Department of Biotechnology, Korea University, Seoul, 02841 Republic of Korea; 2 Department of Food Science and Biotechnology, College of Knowledge-Based Services Engineering, Sungshin Women’s University, Seoul, 01133 Republic of Korea; 3 Department of Food and Nutrition, College of Health & Wellness, Sungshin Women’s University, Seoul, 01133 Republic of Korea

**Keywords:** Biliverdin, *Corynebacterium glutamicum*, In vitro thermodynamic analysis, Coproporphyrin dependent pathway, Synthetic biology, Metabolic engineering

## Abstract

**Background:**

Biliverdin, a prospective recyclable antioxidant and one of the most important precursors for optogenetics, has received growing attention. Biliverdin is currently produced by oxidation of bilirubin from mammalian bile using chemicals. However, unsustainable procedures of extraction, chemical oxidation, and isomer separation have prompted bio-based production using a microbial cell factory.

**Results:**

In vitro thermodynamic analysis was performed to show potential candidates of bottleneck enzymes in the pathway to produce biliverdin. Among the candidates, *hemA* and *hemL* were overexpressed in *Corynebacterium glutamicum* to produce heme, precursor of biliverdin. To increase precursor supply, we suggested a novel *hemQ*-mediated coproporphyrin dependent pathway rather than noted *hemN*-mediated protoporphyrin dependent pathway in *C. glutamicum*. After securing precursors, *hmuO* was overexpressed to pull the carbon flow to produce biliverdin. Through modular optimization using gene rearrangements of *hemA*, *hemL*, *hemQ*, and *hmuO*, engineered *C. glutamicum* BV004 produced 11.38 ± 0.47 mg/L of biliverdin at flask scale. Fed-batch fermentations performed in 5 L bioreactor with minimal medium using glucose as a sole carbon source resulted in the accumulation of 68.74 **±** 4.97 mg/L of biliverdin, the highest titer to date to the best of our knowledge.

**Conclusions:**

We developed an eco-friendly microbial cell factory to produce biliverdin using *C. glutamicum* as a biosystem. Moreover, we suggested that *C. glutamicum* has the thermodynamically favorable coproporphyrin dependent pathway. This study indicated that *C. glutamicum* can work as a powerful platform to produce biliverdin as well as heme-related products based on the rational design with in vitro thermodynamic analysis.

**Electronic supplementary material:**

The online version of this article (10.1186/s13036-019-0156-5) contains supplementary material, which is available to authorized users.

## Background

Biliverdin, a tetrapyrrolic pigment, is a product of heme catabolism that consists of several isomers such as biliverdin IX alpha (α), beta (β), gamma (γ) and delta (δ) [1]. Especially, biliverdin IXα, which is typically known as biliverdin, has been considered to be a novel recyclable antioxidant for humans [2]. In human bodies, glutathione is recycled in the glutathione cycle to protect water-soluble proteins from reactive oxygen species (ROS), and biliverdin protects lipids from ROS as a component of the biliverdin-bilirubin cycle [3]. Biliverdin is gaining interest in many medical fields including research on antiviral agents [4], inflammation regulators [5], and medicine for lung graft injury [6] or liver ischemia reperfusion injury [7]. Moreover, it has been recognized as an important precursor of various chromophores for phytochromes used in material sciences [8], optogenetics [9] and synthetic biology [10].

Current methods to produce biliverdin are related to the chemical oxidation of bilirubin that was extracted from mammalian bile [11, 12]. These methods raised some problems such as environmental disruption, impurity, and isomer production. In contrast to chemical processing, bio-based production using microbial cell factories has been considered as a rational source for the production of medicines, materials, fuels, and a broad range of chemicals [13]. Biliverdin is biologically produced via the heme biosynthesis pathway. Heme, a direct precursor of biliverdin, is an important material for living organisms; therefore, the heme biosynthesis pathway is active in most organisms. The heme biosynthesis pathway is composed of almost ten enzymes in two forks [14]. The first fork aims to produce 5-aminolevulinic acid from amino acids such as glycine or glutamate. One route of the first fork is the C4 pathway that directly condensates glycine and succinyl-CoA in mammals, birds, and fungi [15]. Another route is the C5 pathway, which starts from glutamate using glutamyl-tRNA synthetase, glutamyl-tRNA reductase, and glutamate-1-semialdehyde aminotransferase in most bacteria, archaea and, plants [16]. The second fork aims to produce heme from coproporphyrinogen III via two different pathways. The protoporphyrin dependent pathway uses protoporphyrin IX as a frame for heme, but the recently discovered coproporphyrin dependent pathway utilizes a coproporphyrin III chassis for heme [17].

A few biological production methods of biliverdin using *E. coli* have been suggested, but the yield was low despite the constant addition of nitrogen sources such as yeast extract or peptone [18]. Furthermore, there have been some efforts to produce phycobilins using mammalian cell or *E. coli* but titers were too low due to the low accumulation of biliverdin, the precursor of phycobilins [9, 19, 20]. Thus, it is necessary to develop another strategy for efficient bio-based production of biliverdin. One of the attractive biosystems in synthetic biology is *Corynebacterium glutamicum* which has been used for the industrial production of amino acids such as glutamate or lysine [21]. It has already been recognized as an efficient biosystem to produce various compounds such as chemicals, fuels, and materials for healthcare [22]. Even though *C. glutamicum* had the limelight for production of 5-aminolevulinic acid which is intermediate in biliverdin biosynthesis pathways, studies on the production of other tetrapyrroles were limited [23]. There are some advantages to use *C. glutamicum* which is known to have the C5 pathway and protoporphyrin-dependent pathway for biliverdin production. It naturally produces large amounts of glutamate, the first intermediate of the C5 pathway. Moreover, *C. glutamicum* contains native heme oxygenase which can produce biliverdin from heme [24]. However, there are no enzymes that can produce isomers of biliverdin such that additional separation methods are unnecessary, unlike current approaches [11, 12]. Additionally, it is generally regarded as a safe (GRAS) gram-positive bacteria which is appropriate for producing pharmaceutical products such as biliverdin.

Recently, the rational design of microbial cell factories has become increasingly important due to the abstruseness of the control of carbon flow in microorganisms [25]. Push and pull engineering is one of the methods for the reasonable design of microbial cell factories [26]. Push engineering overexpresses enzymes that act as a bottleneck for pushing carbon flow to produce plenty of precursors, and pull engineering aims to pull carbon flow to targeted final products by strengthening terminal enzymes. This method is commonly based on previous studies about each enzyme related to the pathway, but in vitro thermodynamic analysis has considerable potential as an alternative way to find bottlenecks in the pathway [27, 28]. In vitro thermodynamic analysis calculates the Gibbs free energy of each enzyme in the path and represents hypothetical rate-limiting steps [29].

**Fig. 1:**
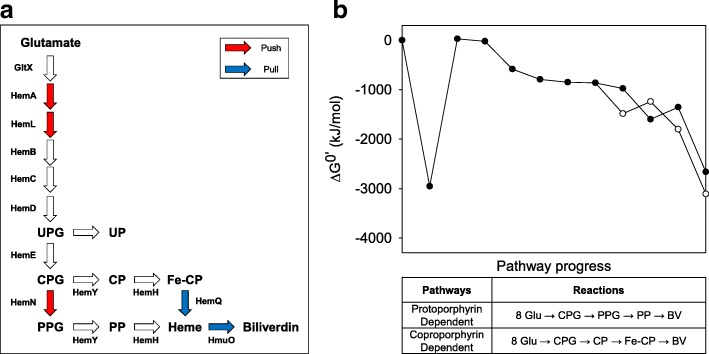
Schematic representation and thermodynamic data of biosynthetic pathway for production of biliverdin. **a** Metabolic pathway and main push and pull strategies for biliverdin biosynthesis in *C. glutamicum*. The red arrows represent the push steps and the blue arrows represent the pull steps. **b** Comparing the changes in Gibbs free energy of reaction as a function of pathway progress from glutamate to biliverdin. Closed circle, protoporphyrin dependent pathway; Open circle, coproporphyrin dependent pathway. Abbreviations: UPG, uroporphyrinogen III; UP, uroporphyrin III; CPG, coproporphyrinogen III; CP, coproporphyrin III, Fe-CP, Coproheme III; PPG, protoporphyrinogen IX; PP, protoporphyrin IX; GltX, glutamyl-tRNA synthetase; HemA, glutamyl-tRNA reductase; HemL, glutamate-1-semialdehyde 2,1-aminomutase; HemB, porphobilinogen synthase; HemC, hydroxymethylbilane synthase; HemD, uroporphyrinogen III synthase; HemE, uroporphyrinogen decarboxylase; HemY, prtoporphyrinogen/coproporphyrinogen III oxidase; HemH, protoporphyrin/coproporphyrin ferrochelatase; HemQ, Fe-coproporphyrin III decarboxylase; HemN, coproporphyrinogen III oxidase; HmuO, heme oxygenase

In this study, we developed *C. glutamicum* platform strains for the efficient production of biliverdin. A push and pull strategy with in vitro thermodynamic analysis was used because of a convoluted and unrevealed heme biosynthesis pathway (Fig. 1a). Push and pull modules were constructed after the investigation of candidate genes. Moreover, a combination of modules through modular optimization and the addition of biotin enabled the production of biliverdin in *C. glutamicum*. Finally, fed-batch fermentations were performed using 5 L bioreactor for the scalable production of biliverdin.

## Results

### Identification of the genes for a push module using in vitro thermodynamic analysis

The heme biosynthesis pathway is known as a tightly regulated pathway and its regulation mechanism is still ambiguous [30]. Moreover, it is hard to accurately quantify the intermediates of this pathway because porphyrinogens easily change to porphyrins due to their ability to autoxidize [31]. To efficiently identify the rate-limiting steps, levels of change in Gibbs free energies in the heme biosynthesis pathway were calculated (Fig. 1b and Table 1). Thermodynamically favorable reactions are exergonic and have negative ΔG^0^′. On the contrary, reactions that have a positive ΔG^0^′ are endergonic and thermodynamically unfavorable. The calculations showed that only *hemA* (glutamyl-tRNA reductase) and *hemH* (protoporphyrin ferrochelatase) had positive ΔG^0^′ in the pathway, and the values were 372.69 kJ/mol and 244.49 kJ/mol, respectively. *hemL* (glutamate-1-semialdehyde aminotransferase) had nearly zero ΔG^0^′ even though other enzymes had highly negative values. The overall pathway of *C. glutamicum* that is known to have a protoporphyrin-dependent pathway using *hemN* (coproporphyrinogen III oxidase) can be written as the reaction below [24]:

**Table 1:** Calculated ΔG^0^′ values for the reaction to produce biliverdin starting from l-glutamate

Enzymes	Reactions (substrates → products)^a^	ΔG^0^’ (kJ/mol)
GltX	l-glutamate + Glu^tRNA^ + ATP → l-glutamyl-Glu^tRNA^ + AMP + PP_i_	− 369.36
HemA	l-glutamyl-Glu^tRNA^ + NADPH + H^+^ → l-glutamate-1-semialdehyde + Glu^tRNA^ + NADP^+^	372.94
HemL	l-glutamate-1-semialdehyde → 5-aminolevulinate	−6.11
HemB	2 5-aminolevulinate → porphobilinogen + 2 H_2_O	− 141.13
HemC	4 porphobilinogen + H_2_O → hydroxymethylbilane + 4 NH_3_	− 207.52
HemD	hydroxymethylbilane → uroporphyrinogen III + H_2_O	−55.13
HemE	uroporphyrinogen III → coproporphyrinogen III + 4 CO_2_	−14.12
HemN	coproporphyrinogen III + 2 AdoMet → protoporphyrinogen IX + 2 5′-dAdo + 2 l-methionine + 2 CO_2_	− 115.27
HemY	protoporphyrinogen IX + 3 O_2_ → protoporphyrin IX + 3 H_2_O_2_	− 622.83
HemH	protoporphyrin + Fe^2+^ → heme + 2 H^+^	244.65
HemY	coproporphyrinogen III + 3 O_2_ → coproporphyrin III + 3 H_2_O_2_	−622.83
HemH	coproporphyrin III + Fe^2+^ → Fe-coproporphyrin III + 2 H^+^	244.65
HemQ	Fe-coproporphyrin III + 2 H_2_O_2_ → heme + 2 CO_2_ + 4 H_2_O	− 562.79
HmuO	heme + 6 Fd_red_ + 3 O_2_ + 6 H^+^ → biliverdin + Fe^2+^ + CO + 6 Fd_ox_ + 3 H_2_O	− 1308.16

^a^Abbreviations: 5′-dAdo, 5′-deoxyadenosine; AdoMet, S-adenosyl-l-methionine; Fd_ox_, oxidized ferredoxin; Fd_red_, reduced ferredoxin

8 l-Glutamate + 2 AdoMet + 8 ATP + 8 NADPH + 6 Fd_red_ + 12 H^+^ + 6 O_2_ → Biliverdin + 2 l-Methionine + 2 5'-dAdo + 8 AMP + 8 PP_i_ + 8 NADP^+^ + 6 Fd_ox_ + 3 H_2_O_2_ + 11 H_2_O + 4 NH_3_ + CO_2_ + CO.

**Fig. 2:**
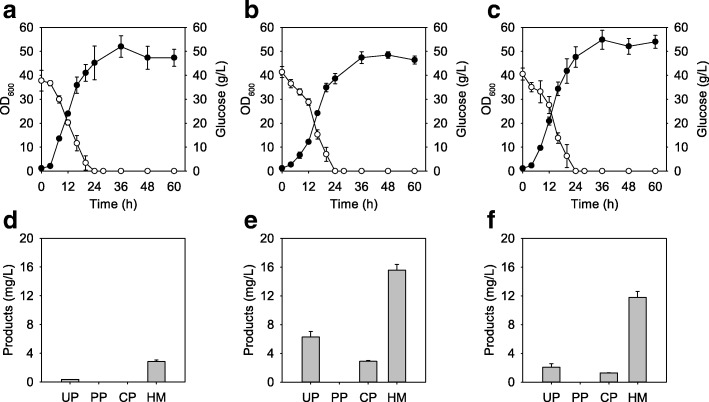
Effect of *hemA*, *hemL*, and *hemN* overexpression as push parts for heme production in *C. glutamicum*. **a**, **b**, and **c** Bacterial cell growth and glucose consumption rate of HM001, HM002, and HM003, respectively. **d**, **e**, and **f** Porphyrin intermediate analysis at 60 h cultivation of HM001, HM002, and HM003, respectively. Closed circles, bacterial cell growth; open circle, residual glucose concentration

The calculated ΔG^0^′ of the overall reaction was − 2661.39 kJ/mol and it was expected to be a highly thermodynamically favorable pathway when enzymes related to bottlenecks were overexpressed (Fig. 1b). Among candidates HemA, HemL, and HemH analyzed through in vitro thermodynamic analysis, HemA and HemL are used eight times to produce biliverdin but HemH used once (Table 1). Based on these data, *hemA* from *Salmonella typhimurium* and *hemL* from *Escherichia coli* were selected as rational components for push modules. Especially, *hemA*^*M*^ (mutated *hemA*) that contained additional lysine near the N-terminus was used for the prevention of the degradation of *hemA* by heme [32]. To identify the effect of overexpression of *hemA*^*M*^ and *hemL*, HM002 strain that contains pEKEx2-hemAL and pMTZ vectors in *C. glutamicum* ATCC 13826, known as a glutamate overproducing strain, was constructed. The HM002 strain showed similar optical density at 60 h, but the specific growth rate was decreased to 0.14 ± 0.01 h^− 1^ compared with 0.24 ± 0.03 h^− 1^ of control strain, HM001 (Fig. 2a-b). Even though HM001 produced little or no porphyrins, HM002 produced 6.29 ± 0.78 mg/L and 2.91 ± 0.11 mg/L of uroporphyrin III (UP) and coproporphyrin III (CP), respectively (Fig. 2d-e). Moreover, the HM002 strain produced 15.59 ± 0.79 mg/L of heme (HM), a 5.48-fold increase compared with control strain HM001. According to the thermodynamic data, we anticipated accumulation of protoporphyrin IX because of the highly positive ΔG^0^′ of *hemH*, which converts protoporphyrin IX to heme (Table 1). However, protoporphyrinogen IX and protoporphyrin IX were not detected.

Therefore, we speculated that overexpression of *hemN* was required to strengthen carbon metabolic flow to protoporphyrin IX for heme production rather than coproporphyrin III, which might not act as a precursor of heme in *C. glutamicum*. Since the amino acid sequences of the *hemN* from *C. glutamicum* ATCC 13826 are the same as that from *C. glutamicum* ATCC 13032, we used the *hemN* from *C. glutamicum* ATCC 13032. As another part for the push module, *hemN* was cloned into a pMTZ vector and expressed in *C. glutamicum* ATCC 13826 with *hemA*^*M*^ and *hemL*. This HM003 strain showed a slightly increased specific growth rate (0.16 ± 0.01 h^− 1^) and optical density at 60 h (54.03 ± 2.73) compared with HM002 (Fig. 2c). However, it could still not produce any protoporphyrinogen IX or protoporphyrin IX. In addition, even heme, uroporphyrin III, and coproporphyrin III were decreased to 11.81 ± 0.83 mg/L, 2.08 ± 0.49 mg/L and 1.30 ± 0.02 mg/L, respectively (Fig. 2f).

### Discovery of *hemQ* as a part for the unrevealed coproporphyrin dependent pathway in *C. glutamicum*

It is generally known that *C. glutamicum* has a protoporphyrin dependent pathway using *hemN*, *hemY* (protoporphyrinogen III oxidase), and *hemH* (Fig. 1a). However, a recent hypothesis that some Actinobacteria and Firmicutes were unable to produce protoporphyrin and used coproporphyrin III as a precursor for heme production has been raised [17]. The coproporphyrin dependent pathway is composed of *hemY*, *hemH*, and *hemQ* (Fe-coproporphyrin III decarboxylase). Differently from their roles in the protoporphyrin-dependent pathway, *hemY* and *hemH* act as coproporphyrinogen III oxidase and coproporphyrin ferrochelatase, respectively, in this pathway. The overall heme biosynthesis pathway that uses coproporphyrin can be written as the reaction below:

8 l-Glutamate + 8 ATP + 8 NADPH + 6 Fd_red_ + 12 H^+^ + 6. O_2_ **→** Biliverdin + 8 AMP + 8 PP_i_ + 8 NADP^+^ + 6 Fd_ox_ + H_2_O_2_ + 15 H_2_O + 4 NH_3_ + 6CO_2_ + CO.

**Fig. 3:**
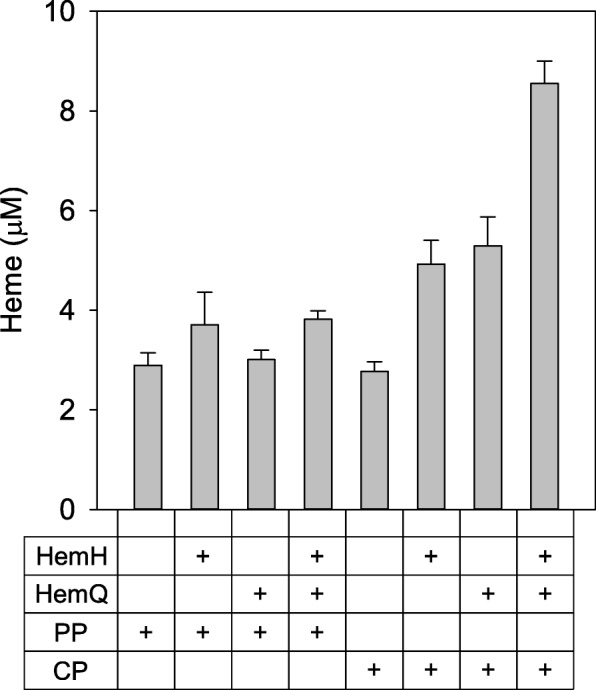
In vitro heme synthesis from protoporphyrin IX or coproporphyrin III using HemH and HemQ from *C. glutamicum*. Plus symbols represent the addition of enzymes (HemH or HemQ) or substrates (protoporphyrin IX or coproporphyrin III)

The ΔG^0^′ of the whole reaction was − 3108.60 kJ/mol and this value was 447.22 kJ/mol less than the value from the protoporphyrin-dependent heme biosynthesis pathway (Fig. 1b and Table 1). Therefore, it was a more thermodynamically favorable reaction and used fewer products such as S-adenosyl-l-methionine. Based on this information, *Cgl1899* in *C. glutamicum* was selected as a putative *hemQ* gene as part of the pull module using BLAST. Crude enzyme extract assays of HemH and HemQ were performed to identify the functional activity of HemQ using HM004, HM005, and HM006 that harboring pMTZ, pMTZ-hemH, and pMTZ-hemQ_NAT_, respectively (Fig. 3). When HemH and HemQ of *C. glutamicum* were assayed with coproporphyrin III, 8.55 ± 0.45 μM of heme, 3.08 times higher than no enzyme expression, was accumulated, confirming that HemQ (Cgl1899) has functional activity. Furthermore, crude extract assays with protoporphyrin IX were performed to compare the protoporphyrin dependent pathway and coproporphyrin dependent pathway. When HemH alone or HemH and HemQ were assayed with protoporphyrin IX, the amount of heme was not significantly increased compared with the results using coproporphyrin III as a substrate. Based on the results, we confirmed the possibility of *hemQ*-mediated coproporphyrin dependent pathway in *C. glutamicum* and *hemQ* was selected as a pull part to produce biliverdin.

**Fig. 4:**
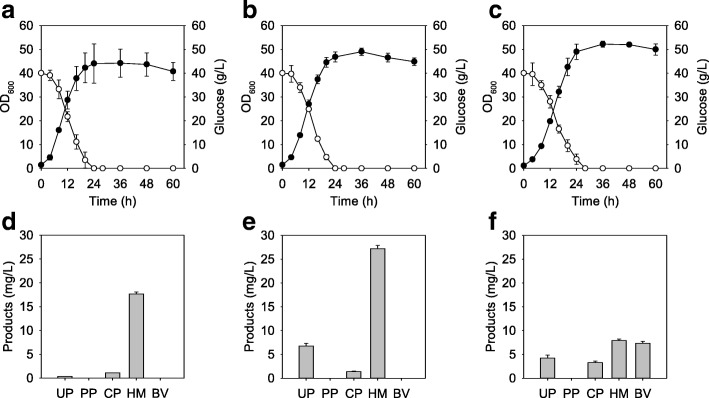
Effect of *hemQ*_NAT_, *hemQ*, and *hmuO* overexpression as pull parts for biliverdin production in *C. glutamicum*. **a**, **b**, and **c** Bacterial cell growth and glucose consumption rate of HM007, HM008, and BV001, respectively. **d**, **e**, and **f** Porphyrin intermediate analysis at 60 h cultivation of HM007, HM008, and BV001, respectively. Closed circles, bacterial cell growth; open circle, residual glucose concentration

To identify the effect of combination of push and pull parts, pMTZ-hemQ_NAT_ carrying the native *hemQ* (*Cgl1899*) gene that uses GTG as a start codon was constructed and cotransformed with pEKEx2-hemAL (named HM007). The HM007 showed a similar growth pattern but the specific growth rate was increased to 0.18 ± 0.01 h^− 1^ compared with HM002 (Fig. 4a). As the *hemQ* gene was added, heme production was increased to 17.66 ± 0.42 mg/L and coproporphyrin III was decreased to 1.14 ± 0.01 mg/L in the HM007 strain (Fig. 4d). To increase HemQ activity, pMTZ-hemQ carrying *Cgl1899* with a change of the GTG start codon to ATG was constructed (named HM008). The HM008 represented a similar growth pattern but produced 27.22 ± 0.65 mg/L of heme, 9.56-fold and 1.74-fold increase compared with HM001 and HM002, respectively (Fig. 4b and e). It produced 1.42 ± 0.07 mg/L of coproporphyrin III and 6.78 ± 0.54 mg/L of uroporphyrin III. However, biliverdin was not detected even though precursors were supplied.

### Enhanced metabolic flux for biliverdin biosynthesis using *hmuO* as a part of the pull module

Because all recombinant strains only produced negligible amounts of biliverdin, the *hmuO* gene from *C. glutamicum* was selected as another part of the pull module and cloned into pMTZ, generating pMTZ-hmuO. The BV001 strain was developed by coexpressing pEKEx2-hemAL and pMTZ-hmuO. During cultivation, the cell growth pattern of the BV001 strain was similar to that of other strains (Fig. 4c). However, it consumed all of the glucose at 28 h while other strains did this at 24 h. During 60 h batch cultivation in a 500 mL baffled flask, 7.37 ± 0.40 mg/L of biliverdin was produced (Fig. 4f). Compared to HM002 that had only a push module, heme which is a direct precursor of biliverdin was decreased to 7.93 ± 0.29 mg/L. Decreased amounts of uroporphyrin III (4.25 ± 0.62 mg/L) and coproporphyrin III (3.27 ± 0.37 mg/L) were also detected. These results showed that the BV001 strain has the potential for production of biliverdin, but more genetic engineering was needed to supply precursors.

**Fig. 5:**
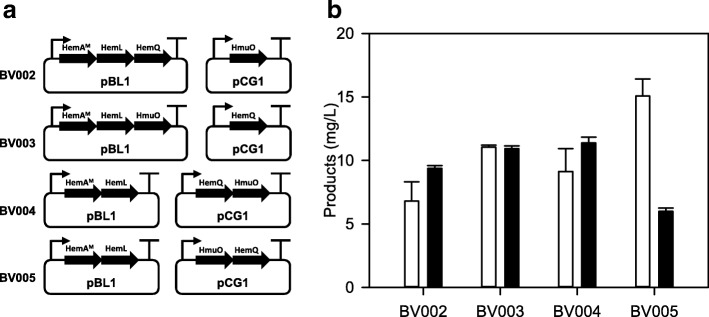
Modular optimization of biliverdin production by combining push module and pull module. **a** Schematic representation of different combinations. The genes *hemA*^M^, *hemL*, *hemQ*, *hmuO* are assembled and rearranged with the plasmids that have different origins. **b** Biliverdin and heme production of recombinant strains with different gene combinations. White box, concentration of heme; black box, concentration of biliverdin

On the basis of the results, we speculated that the utilization of both *hemQ* and *hmuO* as part of the pull module could produce biliverdin more efficiently (Fig. 5a). BV002 harboring pEKEx2-hemALQ and pMTZ-hmuO was constructed and produced 9.39 ± 0.22 mg/L of biliverdin, which was 1.27-fold higher than the BV001 strain (Fig. 5b). Furthermore, another three strains that had both push parts (*hemA*^*M*^ and *hemL*) and pull parts (*hemQ* and *hmuO*) with different genetic arrangements were developed. The BV004 strain harboring pEKEx2-hemAL and pMTZ-hemQO gave better production of biliverdin than the other strains. At 60 h, the BV004 strain produced 11.38 ± 0.47 mg/L of biliverdin, while 9.14 ± 1.79 mg/L of heme remained (Fig. 5b). Meanwhile, 10.93 ± 0.23 mg/L and 6.00 ± 0.27 mg/L of biliverdin were produced in BV003 and BV005, respectively, while 11.04 ± 0.16 mg/L and 15.09 ± 1.32 mg/L of heme had accumulated, respectively. As shown in Additional file 1: Figure S1, four different modules produced different concentrations of uroporphyrin III and coproporphyrin III, but protoporphyrin IX was still not detected.

In addition, the biotin concentration was optimized in BV004 culture for enhanced biliverdin production. Biotin limitation is one of the storage tactics in *C. glutamicum* for the overproduction of glutamate which is an important precursor for biliverdin production. Nevertheless, the concentration of biotin should be optimized because a low level of biotin could inhibit the growth of *C. glutamicum* which is a biotin auxotroph [33]. For the optimization of biotin concentrations, *C. glutamicum* BV004 was cultivated in modified CGX medium with eight different biotin concentrations: 1 μg/L, 5 μg/L, 20 μg/L, 50 μg/L, 100 μg/L, 200 μg/L, 300 μg/L and 500 μg/L (Table 2). BV004 had similar growth patterns when cultivated with biotin concentrations from 20 μg/L to 200 μg/L, and the final OD_600_ reached almost 50. A total of 1 μg/L and 5 μg/L of biotin had a lower final OD_600_ (40.12 ± 0.22 and 49.80 ± 0.63), but 300 μg/L and 500 μg/L of biotin had a final OD_600_ up to almost 59. Among the different concentrations of biotin, BV004 cultivated with 200 μg/L produced the highest biliverdin titer, which was 11.38 ± 0.47 mg/L.

**Table 2:** Biotin optimization for biliverdin production in *C. glutamicum* BV004

Biotin concentration (μg/L)	Cell biomass (OD_600_)	Biliverdin (mg/L)	Biliverdin/cell biomass (OD_600_)
1	40.12 ± 0.22	5.17 ± 1.29	0.13
5	49.80 ± 0.63	7.97 ± 0.33	0.16
20	55.41 ± 0.58	9.88 ± 0.38	0.18
50	55.79 ± 1.11	8.66 ± 0.48	0.16
100	54.00 ± 0.92	7.68 ± 0.61	0.14
200	55.10 ± 0.90	11.38 ± 0.47	0.21
300	59.30 ± 0.92	9.79 ± 0.77	0.17
500	58.93 ± 1.91	8.95 ± 1.46	0.15

*C. glutamicum* BV004 were fermented at 150 rpm for 60 h with 40 g/L initial glucose as a sole carbon source. Results are the means ± standard deviations in three individual experiments

### Biliverdin production by *C. glutamicum* BV004 in fed-batch fermentation

**Fig. 6:**
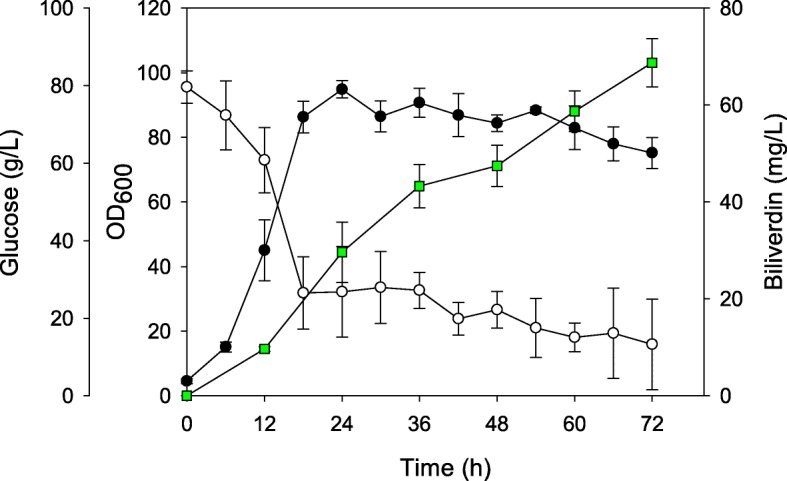
Averaged fed-batch fermentation profile of *C. glutamicum* BV004. The error bars represent the means and standard deviations of triplicate fermentation profiles. Closed circles, bacterial cell growth; open circle, residual glucose concentration; green square, biliverdin concentration

To evaluate the ability of the BV004 strain that produced the highest concentration of biliverdin among all strains, scaled-up fed-batch cultures were carried out in 5 L bioreactor to produce biliverdin (Fig. 6 and Additional file 1: Figure S2). Based on the optimization of the biotin concentration, 200 μg/L of biotin was used in fed-batch fermentation (Table 2). Starting with 80 g/L glucose as the sole carbon source, total 183.02 ± 7.53 g of glucose was consumed. During fed-batch fermentations, 68.74 ± 5.00 mg/L of biliverdin was produced, which is the highest titer using microbial cell factories to the best of our knowledge. The yield and productivity were 0.84 ± 0.07 mg/g glucose and 0.95 ± 0.07 mg/L/h.

## Discussion

Biliverdin has attracted increasing interest due to its wide application in various fields. In particular, it has become apparent that biliverdin is one of the recyclable antioxidants such as glutathione and it is expected that a lower amount of biliverdin was needed for reducing ROS compared to other antioxidants because of its recyclability [2, 3]. In this study, we developed recombinant *C. glutamicum* strains for the efficient production of biliverdin via novel coproporphyrin dependent pathway. Even though *C. glutamicum* is known to have heme oxygenase, biliverdin was not detected during the culture of the wild type strain. Thus, we introduced a rational push and pull strategy with in vitro thermodynamic analysis to produce biliverdin. The strategies used in this study were as follows: (i) overexpression of *hemA*^*M*^ and *hemL* as push parts based on thermodynamic data; (ii) discovery of unnoted *hemQ* as a pull part after comparison of known *hemN* and *hemQ*; (iii) overexpression of *hmuO* as a pull part; (iv) modular optimization of push and pull modules; and (v) fed-batch fermentation to a scalable production of biliverdin using a proper biotin concentration.

The push and pull strategy is one of the logical tactics to produce targeted materials and it has been used in many biosystems such as *Yarrowia lipolytica* [26], *Saccharomyces cerevisiae* [34] and *E. coli* [35]. Briefly, ‘push’ means the overexpression of enzymes that are recognized as bottlenecks in pathways and ‘pull’ means the overexpression of terminal enzymes. This strategy minimizes target genes overexpression for the efficient production of materials. Overexpression of essential enzymes, rather than all of the related enzymes, avoids metabolic burdens and excessive use of antibiotics [36]. Moreover, it is more important in the heme biosynthesis pathway because the enzymes related to this intricately regulated pathway influence the mRNA expression levels of one another [30]. In the case of *E. coli*, the overexpression of *hemD* upregulated almost all of the enzymes in the heme biosynthesis pathway. Conversely, the overexpression of enzymes such as *hemB* or *hemG* reduced the relative mRNA expression levels of other enzymes. Candidates for the components of the push and pull strategy are commonly selected based on many studies on the related enzymes but there were a few articles on the biliverdin biosynthesis pathway in *C. glutamicum*. Therefore, we primally introduced in vitro thermodynamic analysis of all enzymes that were related to the pathway [37, 38]. This analysis provided information on the thermodynamic stability of enzymes using the calculation of Gibbs free energy. Enzymes with higher Gibbs free energies are thermodynamically unfavorable and have greater possibilities to act as rate-limiting steps in pathways [39, 40]. The success of the overexpression of genes based on the calculations in this study corresponded with previous studies that overexpressed *hemA* and *hemL* or *hemH* for heme production [41–43]. Therefore, *hemH* can be a tempting candidate for further studies to enhance heme production in *C. glutamicum*.

The result that the overexpression of *hemN* negatively effects heme production is noteworthy (Fig. 2f). We speculated on the existence of a *hemQ*-mediated pathway because protoporphyrin IX is not detected in *C. glutamicum* despite *hemN* overexpression. The result that *C. glutamicum* does not use protoporphyrin IX but instead uses coproporphyrin III for heme makes *C. glutamicum* a reasonable microbial cell factory to produce materials related to heme. There are some different enzymes related to the protoporphyrin-dependent pathway that use different cofactors [14]. For example, in the case of *E. coli*, *hemF* and *hemG* can replace *hemN* and *hemY*, respectively (Additional file 1: Table S1). There are five methods for producing heme from coproporphyrinogen III. Based on the calculation of the Gibbs free energies, the reaction using *hemY*, *hemH*, and *hemQ*, as with *C. glutamicum,* has the greatest potential among the tested reactions. The ΔG^0^′ of this reaction was − 940.33 kJ/mol, and this value was at least 190.18 kJ/mol less than the other reactions. Moreover, it utilizes the least number of substrates for heme production. It only uses 1 mol coproporphyrinogen III, 3 mol oxygen, and 1 mol ferrous ion to synthesize 1 mol of heme, even though other methods need additional cofactors such as 5′-deoxyadenosines or menaquinones.

Balancing the expression of gene rearrangements may be an important factor in producing materials in synthetic biology [44]. The utilization of substrates or the production of targeted chemicals can be diverse even though the same genes were overexpressed [45, 46]. A pEKEx2 vector that contains a pBL1 origin and a pMTZ vector that carries a pCG1 origin produce moderate copy numbers of plasmids, but a pBL1 origin produces a slightly higher copy number per cell than a pCG1 origin [47]. Four different strains were constructed with different gene arrangements using the pEKEx2 and pMTZ vectors (Fig. 5a). All strains, except for BV005, produced more biliverdin than the BV001 strain. Interestingly, BV005 showed similar production of biliverdin compared to the BV001 strain. It seems that the order of genes is one of the important factors because of various translation levels in a synthetic operon [48]. Modular optimization may be important in the heme biosynthesis pathway because it is tightly regulated and related genes affect each other. Despite using the same genes, such as *hemA*^*M*^, *hemL*, *hemD*, and *hemF*, 5-aminolevulinic acid production was 180 times different depending on modular optimization in *E. coli* [30]. Apart from the biliverdin concentration, uroporphyrin III also seemed to be influenced by gene arrangements. In the heme biosynthesis pathway, uroporphyrinogen III is converted to coproporphyrinogen III by *hemE* or is spontaneously changed to uroporphyrin III without an enzymatic reaction. The amount of accumulated uroporphyrin III is highly affected by the overexpression of genes related to the heme biosynthesis pathway in *E. coli* [49]. When *hemB*, *hemC*, and *hemD* genes were overexpressed in *E. coli*, uroporphyrin III was increased to 4.67-, 2.17-, and 17.5-fold compared to the control strains, respectively. Likewise, the overexpression of *hemQ* with *hmuO* using a moderate copy number vector seems to highly affect the concentration of uroporphyrin III. BV004 and BV005 in which *hemQ* and *hmuO* were overexpressed with the pMTZ vector accumulated almost 20 mg/L of uroporphyrin III, while other strains produced nearly 5 mg/L of uroporphyrin III (Additional file 1: Figure S1). Therefore, *hemE* that strengthens the carbon flow to coproporphyrinogen III and reduces accumulation of uroporphyrin III can be a reasonable candidate for further studies.

## Conclusion

In summary, we suggested the possibility of *C. glutamicum* as a reasonable biosystem to produce biliverdin. We demonstrated the existence of the coproporphyrin dependent pathway in *C. glutamicum* and produced the highest titer of biliverdin using this pathway. Based on this study, further improvements in upstream bioprocessing, such as rerouting the carbon flux of the TCA cycle and introducing a novel pathway to produce more precursors, and downstream bioprocessing, such as developing an efficient purification method, will be necessary for the industrial utilization of biliverdin.

## Methods

### Strains, plasmids, and primers

All bacterial strains and plasmids used in this study are listed in Table 3. All primers used in this study are presented in Table 4. *E. coli* DH5α was used as the host for gene cloning and plasmid construction, and *C. glutamicum* ATCC 13826 was used as the host strain to produce biliverdin. For the two-vector system of *C. glutamicum*, pMTZ vector was constructed by replacing the kanamycin resistance gene of pMT-tac with a zeocin resistance gene from pPsADHα by digestion with SalI and SnaBI. The *hemN*, *hemH*, *hemQ*_NAT_, *hemQ*, and *hmuO* genes were amplified from the genomic DNA of *C. glutamicum*. To construct plasmids pMTZ-hemN, pMTZ-hemH, pMTZ-hemQ_NAT_, pMTZ-hemQ, and pMTZ-hmuO, the *hemN*, *hemH*, *hemQ*_NAT_, *hemQ*, and *hmuO* genes were amplified using primers N-F/N-R, H-F/H-R, Q_NAT_-F/Q_NAT_-R, Q-F/Q-R, and O-F/O-R, respectively. The amplified genes were digested with *Cla*I/*Not*I (*hemN*, *hemH*, *hemQ*_NAT_, and *hemQ*) and *Cla*I/*Bam*HI (*hmuO*) and cloned into plasmid pMTZ. To construct plasmids pEKEx2-hemALQ and pEKEx2-hemALO, *hemQ* and *hmuO* genes were amplified using primers ALQ-F/ALQ-R and ALO-F/ALO-R, respectively. The amplified genes were digested with *Bam*HI/*Kpn*I and cloned into plasmid pEKEx2-hemAL. To construct plasmids pMTZ-hemQO and pMTZ-hemOQ, *hmuO* and *hemQ* genes were amplified using primers QO-F/QO-R and OQ-F/OQ-R, respectively. The amplified genes were digested with *Not*I/*Not*I (*hmuO*) and *Bam*HI/*Not*I (*hemQ*) and cloned into plasmid pMTZ-hemQ and pMTZ-hmuO, respectively.

**Table 3:** Strains and plasmids in this study

Strains and plasmids	Relevant characteristics	References
Strains		
*E. coli* DH5α	F−, *deoR*, *endA1*, *gyrA96*, *hsdR17*(rk − mk+), *recA1*, *relA1*, *supE44*, *thi-1*, Δ (*lacZYA*-*argF*) U169, (Phi80*lacZ*delM15)	Invitrogen
*C. glutamicum* ATCC 13032	Wild type	ATCC
*C. glutamicum* ATCC 13826	Wild type; industrial glutamate producers	ATCC
HM001	*C. glutamicum* ATCC 13826 strain with plasmids pEKEx2 + pMTZ	This work
HM002	*C. glutamicum* ATCC 13826 strain with plasmids pEKEx2-hemAL + pMTZ	This work
HM003	*C. glutamicum* ATCC 13826 strain with plasmids pEKEx2-hemAL + pMTZ-hemN	This work
HM004	*C. glutamicum* ATCC 13826 strain with plasmids pMTZ	This work
HM005	*C. glutamicum* ATCC 13826 strain with plasmids pMTZ-hemH	This work
HM006	*C. glutamicum* ATCC 13826 strain with plasmids pMTZ-hemQ_NAT_	This work
HM007	*C. glutamicum* ATCC 13826 strain with plasmids pEKEx2-hemAL + pMTZ-hemQ_NAT_	This work
HM008	*C. glutamicum* ATCC 13826 strain with plasmids pEKEx2-hemAL + pMTZ-hemQ	This work
HM009	*C. glutamicum* ATCC 13826 strain with plasmids pEKEx2-hemALQ + pMTZ	This work
BV001	*C. glutamicum* ATCC 13826 strain with plasmids pEKEx2-hemAL + pMTZ-hmuO	This work
BV002	*C. glutamicum* ATCC 13826 strain with plasmids pEKEx2-hemALQ + pMTZ-hmuO	This work
BV003	*C. glutamicum* ATCC 13826 strain with plasmids pEKEx2-hemALO + pMTZ-hemQ	This work
BV004	*C. glutamicum* ATCC 13826 strain with plasmids pEKEx2-hemAL + pMTZ-hemQO	This work
BV005	*C. glutamicum* ATCC 13826 strain with plasmids pEKEx2-hemAL + pMTZ-hemOQ	This work
Plasmids		
pPsADHα	Zeo^R^; P_ADH2_, *Pichia pastoris* integration vector	[50]
pEKEx2	Kan^R^; P_tac_, *lacI*^q,^ pBL1 oriV_*C. glutamicum*_; *C. glutamicum* / *E. coli* shuttle vector	[51]
pMT-tac	Kan^R^; P_tac_, *lacI*^q^, pCG1 oriV_*C. glutamicum*_; *C. glutamicum* / *E. coli* shuttle vector	[52]
pMTZ	Zeo^R^; P_tac_, *lacI*^q^, pCG1 oriV_*C. glutamicum*_; *C. glutamicum* / *E. coli* shuttle vector	This work
pEKEx2-hemAL	pEKEx2 carrying *hemA* from *S. typhimurium* and *hemL* from *E. coli*	[41]
pEKEx2-hemALQ	pEKEx2 carrying *hemA* from *S. typhimurium*, *hemL* from *E. coli* and *hemQ* from *C. glutamicum* with an exchange of GTG start codon of *hemQ* to ATG	This work
pEKEx2-hemALO	pEKEx2 carrying *hemA* from *S. typhimurium*, *hemL* from *E. coli* and *hmuO* from *C. glutamicum*	This work
pMTZ-hemN	pMTZ carrying *hemN* from *C. glutamicum*	This work
pMTZ-hemH	pMTZ carrying *hemH* from *C. glutamicum*	This work
pMTZ-hemQ_NAT_	pMTZ carrying *hemQ* from *C. glutamicum*	This work
pMTZ-hemQ	pMTZ carrying *hemQ* from *C. glutamicum* with an exchange of GTG start codon of *hemQ* to ATG	This work
pMTZ-hmuO	pMTZ carrying *hmuO* from *C. glutamicum*	This work
pMTZ-hemQO	pMTZ carrying *hemQ* from *C. glutamicum* with an exchange of GTG start codon of *hemQ* to ATG and *hmuO* from *C. glutamicum*	This work
pMTZ-hemOQ	pMTZ carrying *hmuO* from *C. glutamicum* and *hemQ* from *C. glutamicum* with an exchange of GTG start codon of *hemQ* to ATG	This work

**Table 4:** Primers used in this study

Primers	Sequences (5′ → 3′)^a^
Zeo-F	CCCGTCGACGTTGACAATTAATCATCGGCATAG
Zeo-R	ATTACGTAGTGTCAGTCCTGCTCCTC
ALQ-F	GCCGGATCC**AAGGAGATATAG**ATGAGCGAGCTCGATATTAAACAG
ALQ-R	CGCGGTACCTTAAGGAAGAACCTTAATCAGATCTGC
ALO-F	CCCGGATCC**AAGGAGATATAG**ATGACAAGCATTATTGCAAGCAACAG
ALO-R	CCCGGTACCTTAAGCAAGAGCCTGAAAAACTTGCTG
N-F	GGGATCGATATGTCAGTTTTTGGTGTGTATATTC
N-R	CCCGCGGCCGCTTAGTCTTCTTCACTAAGCAAAATG
H-F	GGGATCGATATGAATGAACGCACATCGGATG
H-R	AAAGCGGCCGCCTAGTTGGCAGCTGGCG
Q_NAT_-F	CCCATCGATGTGAGCGAGCTCGATATTAAAC
Q_NAT_-R	GGGGCGGCCGCTTAAGGAAGAACCTTAATCAGATCTGC
Q-F	CCCATCGATATGAGCGAGCTCGATATTAAACAG
Q-R	CCAGCGGCCGCTTAAGGAAGAACCTTAATCAGATCTGCAATG
O-F	GCGATCGATATGACAAGCATTATTGCAAGC
O-R	GGGGGATCCTTAAGCAAGAGCCTGAAAAACTTGCTGATT
QO-F	CCCGCGGCCGC**AAGGAGATATAG**ATGACAAGCATTATTGCAAGCAA
QO-R	GAGGCGGCCGCTTAAGCAAGAGCCTGAAAAACTTG
OQ-F	CCCGGATCC**AAGGAGATATAG**ATGAGCGAGCTCGATATTAAACAGC
OQ-R	GGGGCGGCCGCTTAAGGAAGAACCTTAATCAGATCTGCAATGT

^a^The underlined letters mean the restriction site and the letters in bold represent the ribosome binding site (RBS) added artificially

### Thermodynamic analysis

The Gibbs free energies for the compounds involved in the pathway to produce biliverdin were obtained from MetaCyC [53]. Net changes in the Gibbs free energy for each reaction were calculated using the equation ΔG^0^′ = Σ(G^0^′_products_) − Σ(G^0^′_substrates_). The values given in kcal/mol were converted to kJ/mol (1 kcal/mol = 4.184 kJ/mol).

### Media composition

LB medium (10 g/L NaCl, 10 g/L tryptone, and 5 g/L yeast extract) was used for molecular genetic procedures. BHIS medium (37 g/L brain heart infusion and 91 g/L sorbitol) was used for the preculture of *C. glutamicum*. Modified CGXII medium consisting of 42 g 3-morpholinopropane-1-sulfonic acid (MOPS), 20 g ammonium sulfate, 5 g urea, 1 g potassium dihydrogen phosphate, 1 g potassium phosphate dibasic anhydrous, 1 g calcium chloride anhydrous, 0.25 g MgSO_4_·7H_2_O, 10 mg CaCl_2_, 10 mg FeSO_4_·7H_2_O, 1 mg ZnSO_4_·7H_2_O, 0.2 mg biotin, 0.31 mg CuSO_4_·5H_2_O, 0.1 mg MnSO_4_·H_2_O and 0.02 mg NiCl_2_·6H_2_O per liter was used for the culture of *C. glutamicum* with glucose as a sole carbon source. When appropriate, kanamycin (50 μg/mL for *E. coli* or 25 μg/mL for *C. glutamicum*), ampicillin (100 μg/mL) or zeocin (25 μg/mL) was added to the medium.

### Flask cultivation

Cells were inoculated into 100 mL baffled flasks containing 20 mL BHIS medium and were cultivated at 30 °C for 12 h with 150 rpm. The precultures were transferred to 500 mL baffled flasks containing 100 mL modified CGXII medium with 40 g/L glucose and 1 mM Isopropyl *β*-d-1-thiogalactopyranoside (IPTG) at an initial OD_600_ of 1. Then, cells were cultivated at 30 °C for 60 h with 150 rpm.

### Fed-batch cultivation

Cells were inoculated into 100 mL baffled flasks containing 20 mL BHIS medium and were cultivated at 30 °C for 12 h with 150 rpm. The first precultures were transferred to 1 L baffled flasks containing 200 mL BHIS medium and were cultivated at 30 °C for 12 h with 150 rpm. The second precultures were washed once with modified CGXII medium and were transferred to 5 L bioreactor containing 1.8 L modified CGXII medium with 80 g/L glucose and 1 mM IPTG. Temperature, agitation, and aeration rate were maintained at 30 °C, 600 rpm, and 2 vvm, respectively. The pH was maintained at 7.0 by automatically adding 10% H_3_PO_4_ and 4 N KOH. When the glucose concentration decreased to approximately 20 g/L, 50% glucose was added to maintain the level of glucose at approximately 20 g/L. Foam was removed using 10% antifoam 204 (Sigma-Aldrich, USA).

### Analytical procedures

Bacterial cell growth was followed by measuring the optical density at 600 nm using a UV/Vis spectrophotometer (Mecasys Co., Ltd., Korea). The glucose concentration in the culture medium was measured with glucose assay kit (Sigma-Aldrich, USA). Porphyrin intermediates and heme concentrations were measured with a high-performance liquid chromatography (HPLC) system (Waters Corporation, USA) with a UV detector (Waters 2487) at 400 nm [41]. The initial mobile phase composition was 20% solvent A (1:9 methanol:acetonitrile) and 80% solvent B (0.5% trifluoroacetic acid in water, pH 2.60). The solvent gradient consisted in 40 min linear change at 95% solvent A and 5% solvent B with 1.0 ml/min flow velocity at 40 °C. Biliverdin concentration was analyzed by HPLC system with a UV detector at 376 nm [54]. The initial mobile phase composition was 25% solvent C (methanol) and 75% (1 M ammonium acetate, pH 5.16). The solvent gradient consisted of 8 min linear change at 95% solvent C and 5% solvent D, and then 2 min at these conditions and 8 min at 25% solvent C and 75% solvent D with 1.5 ml/min flow velocity at 70 °C.

### Enzymatic assays

Cells were cultivated at 30 °C for 16 h with 150 rpm, harvested by centrifugation (4000 rpm at 4 °C for 20 min), washed twice in 66 mM Tris-HCl (pH 8.0). The cells were resuspended in same buffer and sonicated for 20 min. Cell debris was removed by centrifugation (4000 rpm at 4 °C for 20 min) and the supernatant was used to crude extract enzyme assays. The protein concentration was measured by Bradford method [55]. The coupled assays of HemH and HemQ were performed as previously reported [56]. The reactions were performed at 30 °C for 15 min in 66 mM Tris-HCl (pH 8.0) containing 3.3% Tween 20, 5 mM glutathione, 100 μM 2-mercaptoethanol, 100 μM ferrous ammonium sulfate, 25 μM substrate (protoporphyrin IX or coproporphyrin III), and 1 mg/mL crude enzyme extracts.

## Additional file

Additional file 1: **Figure S1.** Production of porphyrin intermediates of recombinant strains with different gene combinations. Black, white and gray bar represent concentration of uroporphyrin III, protoporphyrin IX and coproporphyrin III, respectively. Protoporphyrin IX was not detected in any recombinant strains. **Figure S2.** Non-averaged fed-batch fermentation profiles of *C. glutamicum* BV004 related to the Fig. 6. **a**, **b**, and **c** The first, second, and third rounds of fermentation, respectively. Closed circle, bacterial cell growth; open circle, residual glucose concentration; green square, biliverdin concentration. **Table S1.** Calculated ΔG^0^′ values for the reaction related to heme biosynthesis pathway. (DOCX 419 kb)

## Abbreviations

5′-dAdo: 5′-deoxyadenosine
AdoMet: S-adenosyl-l-methionine
BV: Biliverdin
CP: Coproporphyrin III
CPG: Coproporphyrinogen III
Fd_ox_: Oxidized ferredoxin
Fd_red_: Reduced ferredoxin
Fe-CP: Coproheme III
HM: Heme
PP: Protoporphyrin IX
PPG: Protoporphyrinogen IX
UP: Uroporphyrin III
UPG: Uroporphyrinogen III
