# Supplementary material for: Systems metabolic engineering of Corynebacterium glutamicum for the bioproduction of biliverdin via protoporphyrin independent pathway
Source: J Biol Eng. 2019 Mar 29;13:28. doi: 10.1186/s13036-019-0156-5 (PMC6441180; doi:10.1186/s13036-019-0156-5)
Supplement: Supplementary file 1 — Figure S1. Production of porphyrin intermediates of recombinant strains with different gene combinations. Black, white and gray bar represent concentration of uroporphyrin III, protoporphyrin IX and coproporphyrin III, respectively. Protoporphyrin IX was not detected in any recombinant strains. Figure S2. Non-averaged fed-batch fermentation profiles of C. glutamicum BV004 related to the Fig. 6. a, b, and c The first, second, and third rounds of fermentation, respectively. Closed circle, bacterial cell growth; open circle, residual glucose concentration; green square, biliverdin concentration. Table S1. Calculated ΔG0′ values for the reaction related to heme biosynthesis pathway. (DOCX 419 kb) [file 13036_2019_156_MOESM1_ESM.docx]

**Systems metabolic engineering of *Corynebacterium glutamicum* for the bioproduction of biliverdin via protoporphyrin independent pathway**

Jiho Seok^a^, Young Jin Ko^a^, Myeong-Eun Lee^a^, Jeong Eun Hyeon^a,b,c^, Sung Ok Han^a^#

^a^ Department of Biotechnology, Korea University, Seoul 02841, Republic of Korea

^b^ Department of Food Science and Biotechnology, College of Knowledge-Based Services Engineering, Sungshin Women's University, Seoul 01133, Republic of Korea

^c^ Department of Food and Nutrition, College of Health & Wellness, Sungshin Women's University, Seoul 01133, Republic of Korea

#Corresponding author

Department of Biotechnology, Korea University, Seoul 02841, Republic of Korea

Tel: +82-2-3290-3151

Fax: +82-2-3290-3151

E-mail: samhan@korea.ac.kr


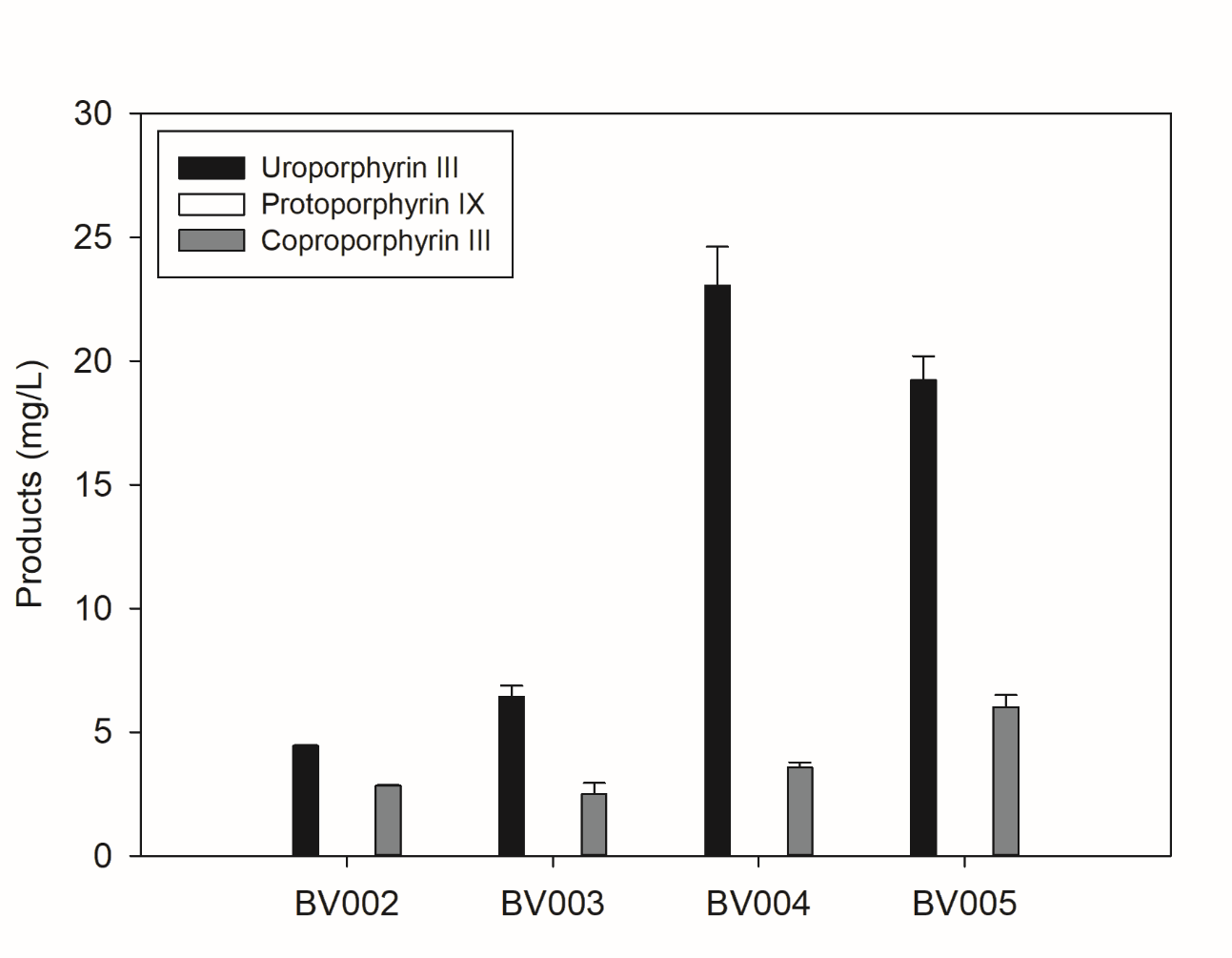


**Figure S1. Production of porphyrin intermediates of recombinant strains with different gene combinations.** Black, white and gray bar represent concentration of uroporphyrin III, protoporphyrin Ⅸ and coproporphyrin III, respectively. Protoporphyrin IX was not detected in any recombinant strains.


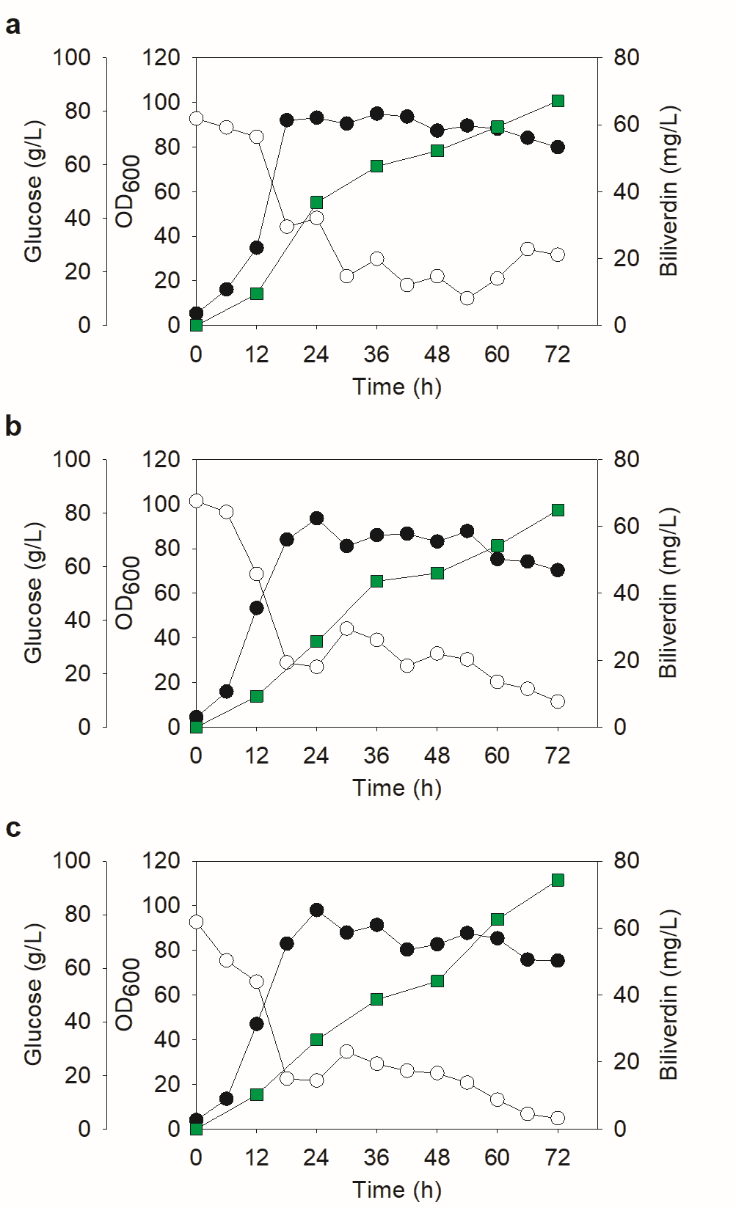


**Figure S2. Non-averaged fed-batch fermentation profiles of *C. glutamicum* BV004 related to the Fig 6.** **a**, **b**, and **c** The first, second, and third rounds of fermentation, respectively. Closed circle, bacterial cell growth; open circle, residual glucose concentration; green square, biliverdin concentration.

**Table S1** Calculated ΔG^0^′ values for the reaction related to heme biosynthesis pathway

| **Enzymes** | **Reactions (substrates → products)** | **ΔG^0^' (kJ/mol)** |
| --- | --- | --- |
| HemF | coproporphyrinogen III + O_2_ + 2 H^+^ → protoporphyrinogen IX + 2 CO_2_ + 2 H_2_O | -372.23 |
| HemG | protoporphyrinogen IX + 3 menaquinone → protoporphyrin IX + 3 menaquinol | -502.19 |
|  |  |  |
| **Related Enzymes** | **Total Reactions (substrates → products)** | **ΔG^0^' (kJ/mol)** |
| HemN  HemY  HemH | coproporphyrinogen III + 2 AdoMet + 3 O_2_ + Fe^2+^  → heme + 2 l-Met + 2 5'-dAdo + 3 H_2_O_2_ + 2H^+^ + 2 CO_2_ | -493.11 |
|  |  |  |
| HemN  HemG  HemH | coproporphyrinogen III + 2 AdoMet + 3 menaquinone + Fe^2+^  → heme + 2 l-Met + 2 5'-dAdo + 3 menaquinol + 2H^+^ + 2 CO_2_ | -372.89 |
|  |  |  |
| HemF  HemY  HemH | coproporphyrinogen III + 4 O_2_ + Fe^2+^  → heme + 3 H_2_O_2_ + 2H_2_O + 2 CO_2_ | -750.15 |
|  |  |  |
| HemF  HemG  HemH | coproporphyrinogen III + 3 menaquinone + O_2_ + Fe^2+^  → heme + 3 menaquinol + 2 H_2_O + 2 CO_2_ | -629.93 |
|  |  |  |
| HemY  HemH  HemQ | coproporphyrinogen III + 3 O_2_ + Fe^2+^  → heme + H_2_O_2_ + 4 H_2_O + 2H^+^ + 2 CO_2_ | -940.33 |
